# The Green Triad: Environmental Knowledge, Nature Connection, and Prosociality as Drivers of Pro-Environmental Behaviour in Chile — A Review

**DOI:** 10.11621/pir.2025.0209

**Published:** 2025-06-01

**Authors:** Alexander Neaman

**Affiliations:** a University of Tarapacá, Arica, Chile

**Keywords:** ecological behaviour, engagement, protection, conservation, environmental education

## Abstract

**Background:**

Addressing global environmental challenges requires an understanding the factors that influence pro-environmental behaviour.

**Objective:**

This review summarises Chilean research that explores how environmental knowledge, nature connection, and prosociality influence such behaviour. The review summarises Chilean studies on these driving factors and discuss their implications for environmental education strategies.

**Design:**

A focussed review was conducted of Chilean studies that employed the General Ecological Behaviour (GEB) scale. This self-reported measure captures a wide range of pro-environmental actions (e.g., recycling, conservation efforts) and environmentally harmful behaviours. The review acknowledges the existence of broader Latin American research on this topic.

**Results:**

The review highlights the importance of specific types of environmental knowledge for pro-environmental behaviour. Knowledge about human impact on ecosystems (human-impact system knowledge) and knowledge of actionable solutions (action knowledge) emerged as key determinants. Interestingly, a knowledge deficit regarding personal effectiveness in environmental protection (effectiveness knowledge) was evident in the Chilean population. In addition, the review emphasises the role of prosocial tendencies, mediated by nature connection, in driving pro-environmental behaviour.

**Conclusion:**

Developing effective environmental education programmes in Chile should prioritise fostering diverse environmental knowledge and strengthening connections with nature. Both environmental knowledge and nature connection can empower individuals and leverage prosocial motivations for positive environmental action. A key limitation of the Chilean studies reviewed are their reliance on cross-sectional data. This type of data allows for the identification of associations but cannot definitively establish causation. To strengthen the causal inferences, future research should employ interventional study designs.

## Introduction

### Review Scope

Environmental degradation is a complex issue intricately linked to human actions (Schultz, 2011). While advancements in technology and legislation are essential, addressing the underlying psychological factors that influence human behaviour is crucial for long-term solutions (Berroeta & Carvalho, 2020; Sandoval-Díaz et al., 2021; Palomo-Vélez et al., 2020). Therefore, it is critical to understand the factors that influence pro-environmental behaviour, defined as actions that minimise environmental harm or actively benefit the environment, such as promoting conservation efforts (Gatersleben, 2019; Masson & Otto, 2021).

Chile currently faces vast and multifaceted environmental issues. These range from habitat destruction and climate change to biodiversity loss and resource scarcity. Unfortunately, environmental issues in Chile are aggravated by social inequality (Solimano & Schaper, 2015). For instance, low-income neighbourhoods in Chile may lack infrastructure such as cycle paths or recycling bins (Berger et al., 2025).

The urgency of addressing environmental challenges in Chile cannot be overstated. Accordingly, it is essential to reveal what motivates the pro-environmental behaviour of individuals within the specific country’s context. Furthermore, previous research on environmental psychology emphasises the specifics of Latin American conditions (Corral-Verdugo & Pinheiro, 2009), thereby justifying the Chilean focus of this review.

**Figure 1:**
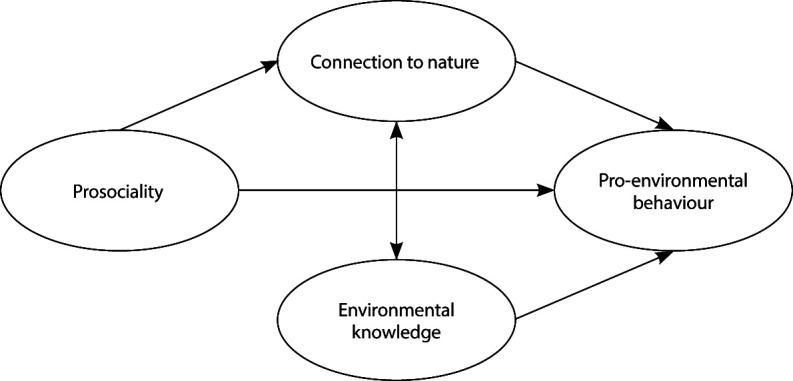
Conceptual model of how environmental knowledge, nature connection, and prosociality influence pro-environmental behaviour

Our review summarises how three key factors — environmental knowledge, nature connection, and prosociality — influence pro-environmental behaviour in Chile’s adult and adolescent populations (*Figure 1*). Environmental knowledge, defined as an individual’s understanding of the environment (Fryxell & Lo, 2003), has been posited as a determinant of pro-environmental behaviour (Geiger et al., 2014; Geiger et al., 2019). Similarly, the role of nature connection in motivating pro-environmental behaviour has been explored in various studies (Barrera-Hernández et al., 2020; Otto & Pensini, 2017). Nature connection, also referred to as “connectedness to nature”, can be defined as the degree to which individuals feel emotionally connected to, and identify with, the natural world (Tanja‐Dijkstra et al., 2019). The concept encompasses various aspects of an individual’s relationship with the natural world, including a feeling of being part of it (Mayer & Frantz, 2004), an appreciation for nature (Brügger et al., 2011), and a sense of closeness to nature (Otto & Pensini, 2017). Finally, prosocial behaviours have traditionally been defined as actions intended to benefit other humans (Eisenberg et al., 2016), which includes acts like sharing, offering comfort, or donating to human-oriented causes (Batson & Powell, 2003; Dunfield, 2014). However, recent research suggests broadening this definition to include the natural world (Duong & Pensini, 2023).

Some researchers propose that pro-environmental behaviour is a form of prosocial behaviour (Kaiser & Byrka, 2011; Kaiser et al., 2015). This perspective suggests that both nature-oriented and human-oriented prosocial behaviours stem from the same underlying prosocial propensity. This refers to an individual’s general inclination to engage in prosocial actions, regardless of whether those actions benefit humans or the environment (Otto et al., 2021). That is to say, individuals with high prosocial propensity are more inclined to take action to help both other humans and the environment. For simplicity, “prosocial propensity” and “prosociality” will be used interchangeably throughout the following discussion.

The research of Otto et al. (2021) proposed that an individual’s prosocial propensity is oriented toward a particular domain of prosocial behaviour (either human or ecological) depending on the individual’s connection to that specific domain (either human or ecological, respectively). The study of Neaman et al. (2022) confirmed this claim by showing that an individual’s prosocial propensity can be actioned into proenvironmental (nature-related) behaviour through connection to nature (*Figure 1*), whereas an individual’s prosocial propensity can be actioned into altruistic (human-related) behaviour through connection to humans.

Thus, our review aims to summarise the existing studies on environmental knowledge, nature connection, and prosociality as motivators of pro-environmental behaviour in Chilean adult and adolescent populations. Furthermore, practical implications on how these drivers can be used for environmental education strategies will be discussed.

### Conceptual Framework: The Campbell Paradigm

A key obstacle to advancing environmental psychology in Latin America is the scarcity of validated scales available in Spanish and Portuguese, to assess pro-environmental behaviours, as identified by Urbina‐Soria & Moyano‐Díaz (2019). This limitation is evident in early Chilean studies (Barazarte et al., 2014; Moyano-Díaz et al., 2017; Otto et al., 2016) that relied on instruments measuring a narrow range of specific actions.

However, Roczen et al. (2014) rightly argue that solely focusing on specific behaviours overlooks the broader spectrum of pro-environmental choices individuals can make. Additionally, individual circumstances significantly influence behavioural opportunities. For example, someone might prioritise energy and water conservation at home over commuting by bicycle due to factors like local infrastructure or personal needs. In essence, life situations and personal choices create a diverse array of pro-environmental possibilities, varying across individuals and contexts.

The limitations of focusing on specific behaviours can be addressed by considering an individual’s general ecological behaviour (Kaiser et al., 2010), which reflects their overall tendency to act pro-environmentally, irrespective of their individual actions. By focusing on this general disposition, the emphasis moves away from specific behaviours and towards an individual’s entire way of life, capturing the degree to which they have adopted a pro-environmental lifestyle.

Gatersleben (2019) identifies the General Ecological Behaviour (GEB) scale by Kaiser & Wilson (2004) as a widely accepted and effective measure of proenvironmental behaviour. This scale assesses a broad spectrum of self-reported actions which focus on protecting nature and minimising environmental impact. Kaiser et al. (2007) proposed an adapted version of the GEB scale for adolescents. The GEB scale’s value is further demonstrated by its successful adaptation for Chilean research - Díaz-Siefer et al. (2015) adapted it for adult populations, while Neaman et al. (2023) focused on adolescents. These adaptations significantly enhance the study of pro-environmental behaviour in Chile across different age groups.

The GEB scales (Kaiser et al., 2007; Kaiser & Wilson, 2004) are rooted in the Campbell Paradigm (Kaiser et al., 2010). This framework builds upon Campbell’s (1963) work and offers an explanation for individual engagement in pro-environmental behaviours. The paradigm focuses on predicting the likelihood of such behaviour, influenced by two key factors:

(1) Environmental motivation: An individual’s overall desire to benefit the environment,
(2) Perceived behavioural difficulty: The ease or difficulty with which a person perceives performing the behaviour. This comprises factors such as time commitment, effort required, financial cost, social norms, and potential social disapproval (Kaiser & Wilson, 2019).

The GEB scales offer a valuable tool for measuring environmental behaviours, categorising them from easy to difficult. This model suggests that individuals with high environmental motivation will likely adopt both easy and difficult behaviours, while those with lower motivation will only engage in easier actions. The paradigm’s strength lies in its simplicity: it allows for a straightforward distinction between individuals with varying degrees of environmental motivation, while encompassing a broad range of behaviours (Gatersleben, 2019).

The Campbell Paradigm’s strength extends beyond pro-environmental behaviour. It can also be applied to assess individual differences in environmental knowledge and nature connection. Similar to behaviour, the paradigm suggests a spectrum for both knowledge and connection, ranging from low to high. Individuals with a strong connection to nature or a high level of environmental knowledge are more likely to engage in a wider range of pro-environmental behaviours (Brügger et al., 2011; Frick et al., 2004). Researchers have successfully adapted existing scales derived from the Campbell Paradigm, including the environmental knowledge scale by Frick et al. (2004) and the nature connection scale by Brügger et al. (2011), to evaluate these factors in their Chilean research.

## Discussion

### Effect of Environmental Knowledge on Pro-Environmental Behaviour

The knowledge-deficit theory (Schultz, 2002) indicates that a lack of knowledge is a key barrier to action. A study by Olivos et al. (2021) supports this theory, demonstrating a positive causal relationship from educational attainment to environmental concern.

Kaiser & Fuhrer (2003) argue that different types of environmental knowledge should be considered to better understand their influence on pro-environmental behaviour. Building on this, Frick et al. (2004) propose three distinct categories of environmental knowledge:

(1) System knowledge (know-what): This encompasses two subcategories: (a) knowledge of how ecosystems function and (b) knowledge of human-caused environmental problems.
(2) Action knowledge (know-how): This refers to knowledge of specific behavioural options and available strategies for reducing human environmental impact.
(3) Effectiveness knowledge: This extends beyond action knowledge by addressing the relative conservational effectiveness associated with a particular behaviour.

Frick et al. (2004) conducted a study to investigate the relationship between the three knowledge types and pro-environmental behaviour. Notably, their findings did not reveal a statistically significant effect of system knowledge on pro-environmental behaviour. Nevertheless, the authors proposed that system knowledge might act as a foundation for other knowledge types. That is to say, a strong understanding of ecosystems and environmental problems (system knowledge) may be necessary for developing knowledge about specific actions (action knowledge) and their effectiveness (effectiveness knowledge). The action and effectiveness knowledge, in turn, are posited to exert a more direct influence on pro-environmental behaviour (*Table 1*).

**Table 1:** The relationship between different environmental knowledge types and pro-environmental behaviour

Type of environmental knowledge	Frick *et al.* (2004)	Díaz-Siefer et al. (2015)
Geography system knowledge	n.s.	Not used
Human-impact system knowledge	Not used	.25
Action knowledge	.12	.22
Effectiveness knowledge	.18	Low reliability

*Note: The Pearson correlation coefficients are shown (p ≤ .001). Geography system knowledge was used in the Swiss study by Frick et al. (2004), whereas human-impact system knowledge was used in the Chilean study by Díaz-Siefer et al. (2015). The effectiveness knowledge scale exhibited low reliability in the latter study and thus was not used in the Pearson correlation analysis. (n.s. = not statistically significant).*

It is important to note a potential limitation in Frick et al.’s (2004) study. Their system knowledge scale focused primarily on geographical knowledge of ecosystem functioning, neglecting another subcategory of system knowledge — the knowledge of human-caused environmental problems.

Building on the three knowledge types proposed by Frick et al. (2004), the Chilean study by Díaz-Siefer et al. (2015) further differentiated the concept of system knowledge. They distinguished between geography system knowledge (i.e., understanding how ecosystems function) and human-impact system knowledge (i.e., knowledge of environmental issues resulting from human actions).

The study by Díaz-Siefer et al. (2015) employed a system knowledge scale exclusively focused on human-impact system knowledge, omitting general geography knowledge. Interestingly, this specific type of system knowledge did exhibit a statistically significant effect on respondents’ pro-environmental behaviour (*Table 1*). The authors suggest that human-impact system knowledge may lead individuals to perceive a connection between their own actions and resulting negative environmental impacts, fostering an “internal locus of control” (Fielding & Head, 2012). This internal sense of agency, where individuals believe their actions can make a difference, is known to promote pro-environmental behaviour.

The findings of Díaz-Siefer et al. (2015) provide valuable insights for designing effective environmental education programmes (Liefländer et al., 2015). Specifically, the study highlights the importance of focussing on the types of knowledge that demonstrably influence pro-environmental behaviour (*Table 1*, that is human-impact system knowledge and action knowledge. In contrast, general knowledge of ecosystem functions (geography system knowledge) may not directly translate into behavioural change (Frick et al., 2004). Furthermore, the research by Díaz-Siefer et al. (2015) revealed a significant knowledge deficit in effectiveness knowledge among the Chilean adult population. Effectiveness knowledge helps individuals choose the most impactful pro-environmental behaviours to implement (Kaiser et al., 2008). Addressing this knowledge gap by educating citizens on the relative environmental benefits of different actions could significantly enhance pro-environmental engagement in Chile.

### Prosociality as a Driver

Recent research suggests a close link between human-oriented prosocial behaviour and pro-environmental behaviour (Neaman et al., 2018), arguing that these behaviours differ only in the beneficiary, that is other humans or the natural world. This aligns with Stern (2000)’s theory, which identifies biospheric and altruistic values as key motivators of human actions. In their study, Stern & Dietz (1994) demonstrated that the biospheric values did not differ from the altruistic values in a general population sample and can thus be combined into biospheric-altruistic values. Therefore, the relation between human-oriented prosocial behaviour and pro-environmental behaviour is consistent with the broader theory by Stern (2000). This means that, values that give substance to a human-oriented prosocial behaviour can be seen as very similar to values related to a pro-environmental behaviour. The role of social values in environmental conservation is also recognised in conservation biology (Manfredo et al., 2017).

Neaman et al. (2018) employed the altruism construct developed by Rushton et al. (1981) as an indicator of prosocial propensity in a Chilean adult population. Their findings revealed a correlation (r = .34) between altruism and pro-environmental behaviour, similar to a study conducted in Mexico (Corral-Verdugo et al. (2011). Notably, Berger & Andaur (2022) found a correlation (r = .26) between human-oriented prosocial behaviour and pro-environmental behaviour in a Chilean adolescent population.

### Nature Connection as a Mediator

A meta-analysis by Whitburn et al. (2020) demonstrates a positive correlation between nature connection and ecological behaviours. Similarly, a review by Steg & Vlek (2009) suggests that, compared to other studied variables, nature connection is a stronger predictor of ecological behaviours. In particular, in a Mexican study of children’s population by Barrera-Hernández et al. (2020), nature connection was identified as a strong positive correlate of pro-environmental behaviour.

Chilean research suggests that nature connection acts as a catalyst, translating prosocial tendencies into pro-environmental actions (Neaman et al., 2023; Neaman et al., 2022; Otto et al., 2021). These studies utilised the HEXACO personality inventory (Lee & Ashton, 2018) to assess prosocial propensity, focusing on the emotionality domain (specifically, sentimentality) and the honesty-humility domain.

According to the HEXACO model of personality, sentimentality refers to the strength of emotional bonds with others and individual differences in empathic sensitivity (Ashton & Lee, 2007), with higher scores indicating greater empathic sensitivity to the feelings of others (www.hexaco.org). Individual differences in empathy have been shown to positively predict altruistic behaviours (Cialdini et al., 1997; Decety et al., 2016; Roberts et al., 2014; Telle & Pfister, 2012). Moreover, individual differences in empathy have also been shown to positively predict ecological behaviours (Brown et al., 2019; Geiger & Keller, 2018; Pfattheicher et al., 2016). Similar to the empathy-altruism hypothesis (Batson et al., 1991; Cialdini et al., 1997), it has been argued that empathy is a pre-requisite for ecological action, proposing an additional empathy-sustainability hypothesis (Brown et al., 2019).

The honesty-humility trait reflects fairness and cooperation in social interactions, implying an underlying prosocial tendency (Ashton & Lee, 2007; Pfattheicher & Böhm, 2018). Supporting the idea of ecological behaviour as prosocial action, a meta-analysis of Soutter et al. (2020) identified a positive relationship between honesty-humility and environmentally friendly behaviours. Similarly, in the Chilean study by Palomo-Vélez et al. (2021), honesty-humility predicted the value individuals assign to the sustainable aspects of different products.

The findings from the aforementioned Chilean studies on both adult and adolescent populations reveal a key mediating role of nature connection. While an individual’s prosocial propensity lays the foundation for pro-environmental behaviour, it is the connection to nature that motivates individuals to translate this propensity into action. This is consistent with Steg & de Groot’s (2019) perspective on the causal pathway, which suggests that “connection to nature” precedes “pro-environmental behaviour”, rather than the other way around.

### Implications for Environmental Education

Given that environmental education aims to promote ecologically responsible lifestyles (Roczen et al., 2014), the following section will suggest the implications of the aforementioned findings for environmental education practices in Chile. Three recent Chilean studies (Neaman et al., 2022; Neaman et al., 2023; Otto et al., 2021) provide compelling evidence for a unified approach to environmental and prosocial education. Although traditionally, these subjects are taught separately, this research suggests that combining them could be more effective.

Environmental education programmes often prioritise knowledge acquisition over fostering a connection with nature (Liefländer, 2015; Otto & Pensini, 2017). Additionally, despite prosociality researchers having developed models to predict pro-environmental behaviour (Kollmuss & Agyeman, 2002), these models rarely translate into educational practices. Furthermore, education for sustainable development (ESD) aims to bridge the gap between society and the environment (Hedefalk et al., 2015), the social aspects typically focus on this connection, rather than explicitly integrating prosociality. The Chilean studies highlight a critical oversight in current ESD programs: the potential to leverage prosocial tendencies to promote environmentally friendly behaviour. Building on the Chilean research by Otto et al. (2021), Neaman et al. (2022), and Neaman et al. (2023), which highlights the link between prosociality, nature connection, and pro-environmental behaviour, this section proposes an innovative approach to environmental education.

Traditionally, environmental education focuses on environmental protection and conservation. However, these Chilean studies suggest that emphasising the creation of prosocial learning environments could be equally important. Prosocial education can potentially strengthen environmental socialisation (Bixler et al., 2014), which could, in turn, transform outdoor experiences with classmates into deeper connections with nature, ultimately fostering more sustainable behaviours. Cuadrado et al. (2017) support this idea by demonstrating that cooperative learning contexts, compared to competitive ones, promote pro-environmental behaviour even among individuals with lower environmental knowledge. In conclusion, integrating prosocial education into environmental education programmes has the potential to substantially increase student engagement in pro-environmental behaviours.

Some scholars advocate for a moral education approach to environmental education (Bai, 2012), however the role of religion remains a complex topic. In Chile, where the Catholic faith is dominant, one might expect it to encourage pro-environmental behaviour through moral influence. However, a Chilean study by Neaman et al. (2021) challenges this assumption. Their findings revealed that, compared to non-religious participants, self-identified Catholics in the study scored lower on measures of nature connection. Moreover, no direct link was found between Catholic identity and pro-environmental behaviour. This is consistent with research by Heckscher (2013) that highlights the historical absence of Christian religious groups in environmental movements. Although the role of religious beliefs in fostering pro-environmental behaviour appears limited, the concept of morality itself warrants further exploration. Berger & Andaur’s (2022) study on Chilean adolescents found a negative association between moral disengagement (i.e., the lack of moral commitment) and pro-environmental behaviour. Notably, this relationship was impacted by peer pro-environmental norms, suggesting a social influence on moral decision-making in this context. Nevertheless, it is important to consider Kaiser et al.’s (2008) argument against solely relying on moral preaching in environmental education, particularly in secular societies. As has been explored throughout this paper, pro-environmental behaviour is driven by various motivations (Kaiser et al., 2017), another one of which is intrinsic motivation. This is where individuals prioritise environmental well-being over personal interests (Otto et al., 2014). Intrinsically motivated individuals are more likely to engage in pro-environmental behaviour autonomously, independent of external pressures like peer norms. Therefore, while moral considerations may influence pro-environmental behaviour, fostering intrinsic motivation offers a potentially more robust and effective approach for environmental education programmes.

## Conclusions and Future Research Needs

This review summarised the current understanding of how environmental knowledge, nature connection, and prosociality influence pro-environmental behaviour in Chile’s adult and adolescent populations. The findings carry significant implications for future environmental education practices. Traditionally, environmental education and prosocial education have been taught as separate subjects. However, the research reviewed here suggests a strong justification for integrating these approaches. A unified educational approach that fosters both environmental knowledge and prosocial tendencies is likely to be more effective in promoting sustainable behaviours. This aligns with the broader goals of creating a society that is not only ecologically sustainable but also socially responsible.

The conceptual model (*Figure 1*) suggests a potential reciprocal relationship between environmental knowledge and nature connection, with evidence from German studies further supporting this idea (Liefländer & Bogner, 2018; Otto & Pensini, 2017; Roczen et al., 2014). For instance, the Chilean study by Neaman et al. (2021) found a positive correlation between knowledge of soil science (a specific environmental knowledge domain) and connection to soil (a specific aspect of nature connection). Individuals with a strong connection to nature may be more motivated to learn about the environment, while increased environmental knowledge may, in turn, foster a deeper appreciation for nature. However, further research is needed to explore this potential reciprocal association in the Chilean population.

A key limitation of the Chilean studies reviewed here are their reliance on cross-sectional data. Although this type of data allows for the identification of associations, it cannot definitively establish causation. To strengthen the causal inferences, future research should employ interventional study designs. For example, Coughlan et al. (2022) demonstrated the effectiveness of targeted interventions in increasing adults’ connection to nature. Similarly, DeVille et al. (2021) found that increased exposure to nature is associated with stronger pro-environmental attitudes and behaviours. Furthermore, the longitudinal study by Liefländer et al. (2015) successfully increased children’s environmental knowledge through a specific intervention. These findings indicate that well-designed interventions have the potential to enhance pro-environmental behaviour.

## References

[ref1] Ashton, M.C., & Lee, K. (2007). Empirical, Theoretical, and Practical Advantages of the HEXACO Model of Personality Structure. Personality and Social Psychology Review, 11, 150–166. 10.1177/1088868306294907 18453460

[ref2] Bai, H. (2012). Reclaiming our moral agency through healing: a call to moral, social, environmental activists. Journal of Moral Education, 41(3), 311–327. 10.1080/03057240.2012.691628

[ref3] Barazarte, R., Neaman, A., Vallejo, F., & García, P. (2014). Environmental knowledge and pro-environmental behavior of high school students, in the Valparaíso Region (Chile). Revista de Educación [Journal of Education], 364, 12–34.

[ref4] Barrera-Hernández, L.F., Sotelo-Castillo, M.A., Echeverría-Castro, S.B., & Tapia-Fonllem, C.O. (2020). Connectedness to Nature: Its Impact on Sustainable Behaviors and Happiness in Children. Frontiers in Psychology, 11, 276. 10.3389/fpsyg.2020.00276 32174866 PMC7054437

[ref5] Batson, C.D., Batson, J.G., Slingsby, J.K., Harrell, K.L., Peekna, H.M., & Todd, R.M. (1991). Empathic joy and the empathy-altruism hypothesis. Journal of Personality and Social Psychology, 61(3), 413–426. 10.1037/0022-3514.61.3.413 1941512

[ref6] Batson, C.D., & Powell, A.A. (2003). Altruism and prosocial behavior. Handbook of psychology, 463–484.

[ref7] Berger, C., & Andaur, A. (2022). Integrating prosocial and proenvironmental behaviors: the role of moral disengagement and peer social norms. Psychology Society & Education, 14(3), 18–28. 10.21071/psye.v14i3.15113

[ref8] Berger, C., Andaur, A., Fábrega, C., & Torres Irribarra, D. (2025). Design and analysis of a scale to measure pro-environmental behaviours among adolescents. PsyEcology. 10.1177/21711976251323536

[ref9] Berroeta, H., & Carvalho, L.P.D. (2020). Environmental-Community Psychology in the Study of Disasters: The Importance of Socio-Spatial Links. Psykhe (Santiago), 29(1), 1–16. 10.7764/psykhe.29.1.1579

[ref10] Bixler, R.D., Joseph, S.L., & Searles, V.M. (2014). Volunteers as products of a zoo conservation education program. The Journal of Environmental Education 45(1), 57–73. 10.1080/00958964.2013.814618

[ref11] Brown, K., Adger, W.N., Devine-Wright, P., Anderies, J.M., Barr, S., Bousquet, F., …, & Quinn, T. (2019). Empathy, place and identity interactions for sustainability. Global Environmental Change-Human and Policy Dimensions, 56, 11–17. 10.1016/j.gloenvcha.2019.03.003

[ref12] Brügger, A., Kaiser, F.G., & Roczen, N. (2011). One for all? Connectedness to nature, inclusion of nature, environmental identity, and implicit association with nature. European Psychologist 16, 324–333. 10.1027/1016-9040/a000032

[ref13] Campbell, D.T. (1963). Social attitudes and other acquired behavioral dispositions. In S. Koch (Ed.), Psychology: A study of a science (Vol. 6, pp. 94–172). McGraw-Hill.

[ref14] Cialdini, R.B., Brown, S.L., Lewis, B.P., Luce, C., & Neuberg, S.L. (1997). Reinterpreting the empathy– altruism relationship: When one into one equals oneness. Journal of Personality and Social Psychology, 73, 481–494. 10.1037/0022-3514.73.3.481 9294898

[ref15] Corral-Verdugo, V., Mireles-Acosta, J., Tapia-Fonllem, C., & Fraijo-Sing, B. (2011). Happiness as correlate of sustainable behavior: A Study of pro-ecological, frugal, equitable and altruistic actions that promote subjective wellbeing. Human Ecology Review, 18(2), 95–104.

[ref16] Corral-Verdugo, V., & Pinheiro, J.Q. (2009). Environmental psychology with a Latin American taste. Journal of Environmental Psychology, 29(3), 366–374. 10.1016/j.jenvp.2008.10.007

[ref17] Coughlan, A., Ross, E., Nikles, D., De Cesare, E., Tran, C., & Pensini, P. (2022). Nature guided imagery: An intervention to increase connectedness to nature. Journal of Environmental Psychology, 80, Article 101759. 10.1016/j.jenvp.2022.101759

[ref18] Cuadrado, E., Tabernero, C., García, R., & Luque, B. (2017). The interactive effect of pro-environmental disciplinary concentration under cooperation versus competition contexts. Environmental Education Research, 23(6), 797–811. 10.1080/13504622.2015.1095860

[ref19] Decety, J., Bartal, I.B., Uzefovsky, F., & Knafo-Noam, A. (2016). Empathy as a driver of prosocial behaviour: highly conserved neurobehavioural mechanisms across species. Philosophical Transactions of the Royal Society B-Biological Sciences, 371(1686), Article 20150077. 10.1098/rstb.2015.0077 PMC4685523 26644596

[ref20] DeVille, N.V., Tomasso, L.P., Stoddard, O.P., Wilt, G.E., Horton, T.H., Wolf, K.L., …, & James, P. (2021). Time Spent in Nature Is Associated with Increased Pro-Environmental Attitudes and Behaviors. International Journal of Environmental Research and Public Health, 18(14), 7498. 10.3390/ijerph18147498 34299948 PMC8305895

[ref21] Díaz-Siefer, P., Neaman, A., Salgado, E., Celis-Diez, J.L., & Otto, S. (2015). Human-environment system knowledge: A correlate of pro-environmental behavior. Sustainability, 7, 15510–15526. 10.3390/su71115510

[ref22] Dunfield, K.A. (2014). A construct divided: prosocial behavior as helping, sharing, and comforting subtypes. Frontiers in Psychology, 5, 958. 10.3389/fpsyg.2014.00958 25228893 PMC4151454

[ref23] Duong, M., & Pensini, P. (2023). The Role of Connectedness in Sustainable Behaviour: A Parallel Mediation Model Examining the Prosocial Foundations of Pro-Environmental Behaviour. Personality and Individual Differences, 209, 112216. 10.1016/j.paid.2023.112216

[ref24] Eisenberg, N., VanSchyndel, S.K., & Spinrad, T.L. (2016). Prosocial Motivation: Inferences From an Opaque Body of Work. Child Development, 87, 1668–1678. 10.1111/cdev.12638 28262941

[ref25] Fielding, K.S., & Head, B.W. (2012). Determinants of young Australians’ environmental actions: the role of responsibility attributions, locus of control, knowledge and attitudes. Environmental Education Research, 18(2), 171–186. 10.1080/13504622.2011.592936

[ref26] Frick, J., Kaiser, F.G., & Wilson, M. (2004). Environmental knowledge and conservation behavior: exploring prevalence and structure in a representative sample. Personality and Individual Differences, 37, 1597–1613. 10.1016/j.paid.2004.02.015

[ref27] Fryxell, G.E., & Lo, C.W.H. (2003). The influence of environmental knowledge and values on managerial behaviours on behalf of the environment: An empirical examination of managers in China. Journal of Business Ethics, 46(1), 45–69. 10.1023/a:1024773012398

[ref28] Gatersleben, B. (2019). Measuring Environmental Behaviour. In L. Steg & J.I.M. de Groot (Eds.), Environmental psychology: An introduction (pp. 157–166). John Wiley & Sons.

[ref29] Geiger, S., Otto, S., & Diaz-Marin, J.S. (2014). A diagnostic environmental knowledge scale for Latin America. PsyEcology, 5, 1–36.

[ref30] Geiger, S.M., Geiger, M., & Wilhelm, O. (2019). Environment-Specific vs. General Knowledge and Their Role in Pro-environmental Behavior. Frontiers in Psychology, 10, 718. 10.3389/fpsyg.2019.00718 31001174 PMC6454026

[ref31] Geiger, S.M., & Keller, J. (2018). Shopping for Clothes and Sensitivity to the Suffering of Others: The Role of Compassion and Values in Sustainable Fashion Consumption. Environment and Behavior, 50(10), 1119–1144. 10.1177/0013916517732109

[ref32] Heckscher, J.J. (2013). A “tradition” that never existed: Orthodox Christianity and the Failure of Environmental History. In J. Chryssavgis & B.V. Foltz (Eds.), Toward an Ecology of Transfiguration: Orthodox Christian Perspectives on Environment, Nature, and Creation (pp. 136–151). Fordham University Press. 10.1515/9780823252343-014

[ref33] Hedefalk, M., Almqvist, J., & Ostman, L. (2015). Education for sustainable development in early childhood education: a review of the research literature. Environmental Education Research, 21(7), 975–990. 10.1080/13504622.2014.971716

[ref34] Kaiser, F.G., & Byrka, K. (2011). Environmentalism as a trait: Gauging people’s prosocial personality in terms of environmental engagement. International Journal of Phychology, 46, 71–79. 10.1080/00207594.2010.516830 22044135

[ref35] Kaiser, F.G., Byrka, K., & Hartig, T. (2010). Reviving Campbell’s paradigm for attitude research. Personality and Social Psychology Review, 14, 351–367. 10.1177/1088868310366452 20435803

[ref36] Kaiser, F.G., & Fuhrer, U. (2003). Ecological behavior’s dependency on different forms of knowledge. Applied Psychology: An International Review, 52, 598–613. 10.1111/1464-0597.00153

[ref37] Kaiser, F.G., Kibbe, A., & Arnold, O. (2017). Self-Determined, Enduring, Ecologically Sustainable Ways of Life: Attitude as a Measure of Individuals’ Intrinsic Motivation. In G. Fleury-Bahi, E. Pol, & O. Navarro (Eds.), Handbook of Environmental Psychology and Quality of Life Research (pp. 185–195). Springer. 10.1007/978-3-319-31416-7_10

[ref38] Kaiser, F.G., Oerke, B., & Bogner, F.W. (2007). Behavior-based environmental attitude: Development of an instrument for adolescents. Journal of Environmental Psychology, 27, 242–251. 10.1016/j.jenvp.2007.06.004

[ref39] Kaiser, F.G., Otto, S., & Schuler, J. (2015). Prosocial propensity bias in experimental research on helping behavior: The proposition of a discomforting hypothesis. Comprehensive Psychology, 4, 11. 10.2466/49.CP.4.11

[ref40] Kaiser, F.G., Roczen, N., & Bogner, F.X. (2008). Competence formation in environmental education: Advancing ecology-specific rather than general abilities. Umweltpsychologie, 12, 56–70. 10.5167/uzh-9249

[ref41] Kaiser, F.G., & Wilson, M.R. (2004). Goal-directed conservation behavior: The specific composition of a general performance. Personality and Individual Differences, 36, 1531–1544. 10.1016/j.paid.2003.06.003

[ref42] Kaiser, F.G., & Wilson, M. (2019). The Campbell Paradigm as a Behavior-Predictive Reinterpretation of the Classical Tripartite Model of Attitudes. European Psychologist, 24(4), 359–374. 10.1027/1016-9040/a000364 32116425 PMC7039345

[ref43] Kollmuss, A., & Agyeman, J. (2002). Mind the gap: why do people act environmentally and what are the barriers to pro-environmental behavior? Environmental Education Research, 8, 239–260. 10.1080/13504620220145401

[ref44] Lee, K., & Ashton, M.C. (2018). Psychometric Properties of the HEXACO-100. Assessment, 25(5), 543–556. 10.1177/1073191116659134 27411678

[ref45] Liefländer, A.K. (2015). Effectiveness of environmental education on water: connectedness to nature, environmental attitudes and environmental knowledge. Environmental Education Research, 21(1), 145–146. 10.1080/13504622.2014.927831

[ref46] Liefländer, A.K., & Bogner, F.X. (2018). Educational impact on the relationship of environmental knowledge and attitudes. Environmental Education Research, 24(4), 611–624. 10.1080/13504622.2016.1188265

[ref47] Liefländer, A.K., Bogner, F.X., Kibbe, A., & Kaiser, F.G. (2015). Evaluating environmental knowledge dimension convergence to assess educational programme effectiveness. International Journal of Science Education, 37(4), 684–702. 10.1080/09500693.2015.1010628

[ref48] Manfredo, M.J., Bruskotter, J.T., Teel, T.L., Fulton, D., Schwartz, S.H., Arlinghaus, R., …, & Sullivan, L. (2017). Why social values cannot be changed for the sake of conservation. Conservation Biology, 31(4), 772–780. 10.1111/cobi.12855 27757996

[ref49] Masson, T., & Otto, S. (2021). Explaining the difference between the predictive power of value orientations and self-determined motivation for proenvironmental behavior. Journal of Environmental Psychology, 73, 101555. 10.1016/j.jenvp.2021.101555

[ref50] Mayer, F.S., & Frantz, C.M.P. (2004). The connectedness to nature scale: A measure of individuals’ feeling in community with nature. Journal of Environmental Psychology, 24, 503–515. 10.1016/j.jenvp.2004.10.001

[ref51] Moyano-Díaz, E., Palomo-Vélez, G., Olivos, P., & Sepúlveda-Fuentes, J. (2017). Natural and urban environments determining environmental beliefs and behaviours, economic thought and happiness. Psyecology, 8(1), 75–106. 10.1080/21711976.2016.1272875

[ref52] Neaman, A., Diaz-Siefer, P., Burnham, E., Castro, M., Zabel, S., Dovletyarova, E. A., …, & Otto, S. (2021). Catholic religious identity, prosocial and pro-environmental behaviors, and connectedness to nature in Chile. Gaia-Ecological Perspectives for Science and Society, 30(1), 44–50. 10.14512/gaia.30.1.9

[ref53] Neaman, A., Montero, E., Pensini, P., Burnham, E., Castro, M., Ermakov, D.S., & Navarro-Villarroel, C. (2023). Unleashing the Power of Connection: How Adolescents’ Prosocial Propensity Drives Ecological and Altruistic Behaviours. Sustainability, 15(10), 8070. 10.3390/su15108070

[ref54] Neaman, A., Otto, S., & Vinokur, E. (2018). Toward an integrated approach to environmental and pro-social education. Sustainability, 10, 583–594. 10.3390/su10030583

[ref55] Neaman, A., Pensini, P., Zabel, S., Otto, S., Ermakov, D.S., Dovletyarova, E.A., …, & Navarro-Villarroel, C. (2022). The Prosocial Driver of Ecological Behavior: The Need for an Integrated Approach to Prosocial and Environmental Education. Sustainability, 14(7), 4202, Article 4202. 10.3390/su14074202

[ref56] Neaman, A., Stange, C., Zabel, S., Minkina, T.M., Yanez, C., Burnham, E., & Otto, S. (2021). Teaching soil science: The impact of laboratory and field components on the knowledge and attitude toward soil. Revista Brasileira de Ciencia do Solo [Brazilian Journal of Soil Science], 45, e0210040. 10.36783/18069657rbcs20210040

[ref57] Olivos, F., Palomo-Vélez, G., Olivos-Jara, P., & Liu, M.H. (2021). Educational attainment and environmental concern in China: An instrumental variable approach. Asian Journal of Social Psychology, 24(2), 156–168. 10.1111/ajsp.12431

[ref58] Otto, S., Kaiser, F.G., & Arnold, O. (2014). The Critical Challenge of Climate Change for Psychology Preventing Rebound and Promoting More Individual Irrationality. European Psychologist, 19(2), 96–106. 10.1027/1016-9040/a000182

[ref59] Otto, S., Neaman, A., Richards, B., & Marió, A. (2016). Explaining the ambiguous relations between income, environmental knowledge, and environmentally significant behavior. Society & Natural Resources, 29, 628–632. 10.1080/08941920.2015.1037410

[ref60] Otto, S., & Pensini, P. (2017). Nature-based environmental education of children: Environmental knowledge and connectedness to nature, together, are related to ecological behaviour. Global Environmental Change, 47, 88–94. 10.1016/j.gloenvcha.2017.09.009

[ref61] Otto, S., Pensini, P., Zabel, S., Diaz-Siefer, P., Burnham, E., Navarro-Villarroel, C., & Neaman, A. (2021). The prosocial origin of sustainable behavior: A case study in the ecological domain. Global Environmental Change-Human and Policy Dimensions, 69, 102312. 10.1016/j.gloenvcha.2021.102312

[ref62] Palomo-Vélez, G., Buczny, J., & Van Vugt, M. (2020). Encouraging Pro-Environmental Behaviors Through Children-Based Appeals: A Kin Selection Perspective. Sustainability, 12(2), 748. 10.3390/su12020748

[ref63] Palomo-Vélez, G., Perlaviciute, G., Contzen, N., & Steg, L. (2021). Promoting energy sources as environmentally friendly: does it increase public acceptability? Environmental Research Communications, 3(11), Article 115004. 10.1088/2515-7620/ac32a8

[ref64] Pfattheicher, S., & Böhm, R. (2018). Honesty-humility under threat: Self-uncertainty destroys trust among the nice guys. Journal of Personality and Social Psychology, 114, 179–194. 10.1037/pspp0000144 28263618

[ref65] Pfattheicher, S., Sassenrath, C., & Schindler, S. (2016). Feelings for the Suffering of Others and the Environment: Compassion Fosters Proenvironmental Tendencies. Environment and Behavior, 48(7), 929–945. 10.1177/0013916515574549

[ref66] Roberts, W., Strayer, J., & Denham, S. (2014). Empathy, Anger, Guilt: Emotions and Prosocial Behaviour. Canadian Journal of Behavioural Science-Revue Canadienne des Sciences du Comportement, 46(4), 465–474. 10.1037/a0035057

[ref67] Roczen, N., Kaiser, F.G., Bogner, F.X., & Wilson, M. (2014). A competence model for environmental education. Environment and Behavior, 46(8), 972–992. 10.1177/0013916513492416

[ref68] Rushton, J.P., Chrisjohn, R.D., & Fekken, G.C. (1981). The altruistic personality and the self-report altruism scale. Personality and Individual Differences, 2, 293–302. 10.1016/0191-8869(81)90084-2

[ref69] Sandoval-Díaz, J., Neumann, P., & Clericus, R.R. (2021). Preliminary Adaptation and Validation of the Cultural Theory of Environmental Worldviews Scale in Chilean Population. Revista Ces Psicologia, 14(1), 16–35. 10.21615/cesp.14.1.3

[ref70] Schultz, P.W. (2002). Knowledge, information, and household recycling: Examining the knowledge-deficit model of behavior change. In T. Dietz & P.C. Stern (Eds.), New tools for environmental protection: Education, information, and voluntary measures (pp. 67–82). National Academy Press.

[ref71] Schultz, P.W. (2011). Conservation Means Behavior. Conservation Biology, 25(6), 1080–1083. 10.1111/j.1523-1739.2011.01766.x 22070255

[ref72] Solimano, A., & Schaper, M. (2015). The paradoxes of Chilean economic development: Growth, inequality, deindustrialisation and sustainability risks. In A. Hansen & U. Wethal (Eds.), Emerging Economies and Challenges to Sustainability: Theories, strategies, local realities (pp. 162–176). Routledge. 10.4324/97811315797106

[ref73] Soutter, A., Bates, T., & Mottus, R. (2020). Big Five and HEXACO Personality Traits, pro-environmentalattitudes, and behaviors: A meta-analysis. Perspectives on Psychological Science. 10.1177/1745691620903019 PMC7333518 32384257

[ref74] Steg, L., & de Groot, J.I.M. (2019). Environmental psychology: An introduction. John Wiley & Sons.

[ref75] Steg, L., & Vlek, C. (2009). Encouraging pro-environmental behaviour: An integrative review and research agenda. Journal of Environmental Psychology, 29, 309–317. 10.1016/j.jenvp.2008.10.004

[ref76] Stern, P., & Dietz, T. (1994). The value basis of environmental concern Journal of Social Issues, 50, 65–84.

[ref77] Stern, P.C. (2000). Toward a coherent theory of environmentally significant behavior. Journal of Social Issues, 56(3), 407–424.

[ref78] Tanja‐Dijkstra, K., Maas, J., van Dijk‐Wesselius, J., & van den Berg, A. (2019). Children and the natural environment. In L. Steg & J. I. M. de Groot (Eds.), Environmental psychology: An introduction (pp. 95–103). John Wiley & Sons.

[ref79] Telle, N.T., & Pfister, H.R. (2012). Not Only the Miserable Receive Help: Empathy Promotes Prosocial Behaviour Toward the Happy. Current Psychology, 31(4), 393–413. 10.1007/s12144-012-9157-y

[ref80] Urbina‐Soria, J., & Moyano‐Díaz, E. (2019). Environmental psychology in Latin America. In L. Steg & J.I.M. de Groot (Eds.), Environmental psychology: An introduction (pp. 251–259). John Wiley & Sons.

[ref81] Whitburn, J., Linklater, W., & Abrahamse, W. (2020). Meta‐analysis of human connection to nature and proenvironmental behavior. Conservation Biology, 34(1), 180–193. 10.1111/cobi.13381 31251416 PMC7027494

